# Evaluation of the Implementation of FDG-PET/CT and Staging Laparoscopy for Gastric Cancer in The Netherlands

**DOI:** 10.1245/s10434-020-09096-z

**Published:** 2020-09-08

**Authors:** Emma C. Gertsen, Alicia S. Borggreve, Hylke J. F. Brenkman, Rob H. A. Verhoeven, Erik Vegt, Richard van Hillegersberg, Peter D. Siersema, Jelle P. Ruurda, on behalf of the Dutch Upper Gastrointestinal Cancer Audit (DUCA) Group

**Affiliations:** 1 Department of Surgery, University Medical Center Utrecht, Utrecht University, Utrecht, The Netherlands; 2 Department of Radiation Oncology, University Medical Center Utrecht, Utrecht University, Utrecht, The Netherlands; 3 Department of Research and Development, Netherlands Comprehensive Cancer Organization (IKNL), Utrecht, The Netherlands; 4 Department of Radiology and Nuclear Medicine, Erasmus University Medical Center Rotterdam, Rotterdam, The Netherlands; 5 Department of Nuclear Medicine, The Netherlands Cancer Institute, Amsterdam, The Netherlands; 6 Department of Gastroenterology and Hepatology, Radboud University Medical Center, Nijmegen, The Netherlands; 7 Department of Surgery, Radboud University Medical Center, Nijmegen, The Netherlands; 8 Department of Surgery, Division Cancer Center, University Medical Center Utrecht, Utrecht, The Netherlands

## Abstract

**Background:**

The role of ^18^F-fluorodeoxyglucose positron emission tomography with computed tomography (FDG-PET/CT) and staging laparoscopy (SL) has increased in the preoperative staging of gastric cancer. Dutch national guidelines have recommended the use of FDG-PET/CT and SL for patients with locally advanced tumors since July 2016.

**Objective:**

The aim of this study was to evaluate the implementation of FDG-PET/CT and SL in The Netherlands.

**Methods:**

Between 2011 and 2018, all patients who underwent surgery for gastric cancer were included from the Dutch Upper GI Cancer Audit. The use of FDG-PET/CT and SL was evaluated before and after revision of the Dutch guidelines. Outcomes included the number of non-curative procedures (e.g. palliative and futile procedures) and the association of FDG-PET/CT and SL, with waiting times from diagnosis to the start of treatment.

**Results:**

A total of 3310 patients were analyzed. After July 2016, the use of FDG-PET/CT (23% vs. 61%; *p* < 0.001) and SL (21% vs. 58%; *p* < 0.001) increased. FDG-PET/CT was associated with additional waiting time to neoadjuvant therapy (4 days), as well as primary surgical treatment (20 days), and SL was associated with 8 additional days of waiting time to neoadjuvant therapy. Performing SL or both modalities consecutively in patients in whom it was indicated was not associated with the number of non-curative procedures.

**Conclusion:**

During implementation of FDG-PET/CT and SL after revision of the guidelines, both have increasingly been used in The Netherlands. The addition of these staging methods was associated with increased waiting time to treatment. The number of non-curative procedures did not differ after performing none, solely one, or both staging modalities.

For patients with locally advanced gastric cancer, the main curative treatment comprises perioperative chemotherapy and gastrectomy.^1^^–^3 The standard initial staging of gastric cancer consists of gastroscopy and computed tomography (CT) of the thorax and abdomen.^4^ However, these modalities frequently miss distant metastases or tumor invasion in adjacent structures,^5^^–^7 which are important characteristics that limit curative treatment. As a result, patients may undergo neoadjuvant chemotherapy and/or surgery without any evident survival benefit, but with the risk of additional morbidity and short-term mortality, due to surgery as well as chemotherapeutic toxicity.

There has been an increasing interest in the role of fluorodeoxyglucose positron emission tomography with CT (FDG-PET/CT) and staging laparoscopy (SL) in the preoperative staging of gastric cancer. Compared with CT alone, FDG-PET/CT has been reported to detect additional distant metastases in 10% of patients with locally advanced gastric cancer, whereas SL detects peritoneal metastases in another 19% of patients.^8^ If distant metastases are detected during the diagnostic process, a more tailored treatment can be offered, such as systemic treatment with palliative intent. In July 2016, the Dutch national guidelines for the diagnosis and treatment of gastric cancer have been revised and advise FDG-PET/CT and SL for patients with locally advanced tumors that are considered for treatment with curative intent^1^; however, the consequences of these new guidelines on patient outcomes are not yet clear. The main potential positive effect is reduction of non-curative procedures, whereas the main possible negative effect is delay of treatment, which is undesirable from a patient perspective. The aim of the current population-based study was to evaluate the implementation of FDG-PET/CT and SL in The Netherlands and its effect on non-curative resection rates and waiting time from diagnosis to treatment.

## Methods

### Study Design

This population-based observational study retrieved anonymous data from the Dutch Upper GI Cancer Audit (DUCA) database. DUCA is a national surgical registry of all patients who underwent surgery for gastroesophageal cancer since 2011. Patients in whom no surgical procedure was performed, for example due to distant metastases detected by FDG-PET/CT, are not registered in the DUCA database. For Dutch hospitals performing gastroesophageal cancer surgery, it is mandatory to provide patient-, tumor- and surgical treatment-related data to the DUCA every year, which is part of the Dutch Institute for Clinical Auditing. An in-depth quality investigation of this national audit has shown trustworthy and complete data registry.^9^ The current study was approved by the Scientific Committee of DUCA, and no ethical approval or informed consent was required according to Dutch law.

### Study Population

All patients who underwent any type of surgery for gastric adenocarcinoma between 2011 and 2018 in The Netherlands were included. Patients with inadequate staging (no diagnostic CT scan), or who underwent emergency, prophylactic or other resection, other than gastrectomy, or with missing data preventing the analysis of the study outcomes (e.g. time of diagnosis), were excluded.

### Diagnosis and Treatment

In The Netherlands, the diagnosis, staging, and treatment of gastric cancer is advised to be performed according to Dutch national guidelines and the 7th edition of the American Joint Committee on Cancer TNM staging system.^1^,^4^,^10^

Centralization of gastric cancer surgery has been gradually introduced in The Netherlands during the study period. As of 2013, a minimum of 20 gastrectomies per center per year is required^9^,^11^,^12^; a center performing at least that number of resections in a year is defined as a high-volume center. The recommended staging process consists of gastroscopy with biopsies and CT scan of the thorax and abdomen in all patients. If there is doubt about the depth of ingrowth, an endoscopic ultrasound (EUS) can be performed to make a better distinction between cT1-2 and cT3-4, or to decide on whether or not to perform an endoscopic mucosal resection (EMR)/endoscopic submucosal dissection (ESD). Since July 2016, FDG-PET/CT and SL with peritoneal lavage and cytology are advised by the Dutch national guidelines for patients with a locally advanced tumor detected on CT (FDG-PET/CT in patients with ≥ cT3 and/or cN + tumors, and SL in patients with ≥ cT3 tumors).^1^ If no metastases are diagnosed, the recommended curative treatment consists of surgical resection by (sub)total gastrectomy with lymphadenectomy according to the Japanese Gastric Cancer Treatment Guidelines.^13^ All patients with resectable gastric cancer (clinical stage > I) are treated with perioperative chemotherapy similar or comparable to the MAGIC or FLOT4 trials^2^,^3^,^14^ if deemed fit enough. Palliative treatment consists of systemic chemotherapy and palliative resection or radiotherapy in patients with symptoms, such as obstruction or bleeding.

### Study Outcomes

The study outcomes included adherence to the national guidelines before and after publication of the new update of the guidelines (1 July 2016), waiting time from diagnosis to the start of treatment, and number of non-curative procedures. Treatment in adherence to the revised guidelines was defined as the proportion of patients who underwent FDG-PET/CT or SL who had an indication for these diagnostic modalities as stated earlier, and the proportion of patients not undergoing these modalities if there was no indication. To evaluate waiting time, the time of diagnosis was defined as the date of the pathology report of the endoscopic biopsies confirming the presence of gastric cancer, and the time of treatment was defined as either the start date of neoadjuvant therapy or the date of surgery in case of primary surgery. Since SL in clinical practice was frequently performed during the same procedure as the planned gastrectomy, it was decided not to perform an analysis on waiting time after SL for the group who underwent primary surgery. Non-curative procedures consisted of palliative gastrectomy (which was intended to be curative before the start of the procedure), construction of a bypass (i.e. no resection), or a futile procedure. In order to analyze the effects of FDG-PET/CT and SL on the rate of non-curative procedures, a subselection was made, including all patients with curative intent with at least a ≥ cT3 and/or N + tumor, as this is the indication for performing FDG-PET/CT (≥ cT3 and/or N +) or SL (≥ cT3) according to the current Dutch guidelines.

### Statistical Analysis

Patient-, tumor-, and treatment-related characteristics were evaluated and described, as was the frequency of missing values per variable. Missing values in time points to evaluate waiting time were imputed using means of the total cohort and other known time points during diagnostic work-up and treatment. Baseline characteristics were compared between patients undergoing or not undergoing FDG-PET/CT or SL, using the Chi square test, Student’s *t* test, or Mann–Whitney U test, depending on type and distribution of the variable. Waiting time from diagnosis to the start of treatment was visually inspected, and, because of a non-normal distribution, was logarithmically transformed before performing univariable and multivariable linear regression analyses. In order to determine the differences in the proportion of non-curative procedures with or without FDG-PET/CT or SL, cross tables with Chi square statistics were generated. All statistical analyses were performed using SPSS version 25.0 (IBM Corporation, Armonk, NY, USA). Statistical significance was set at *p* < 0.05.

## Results

### Study Population

**Fig. 1:**
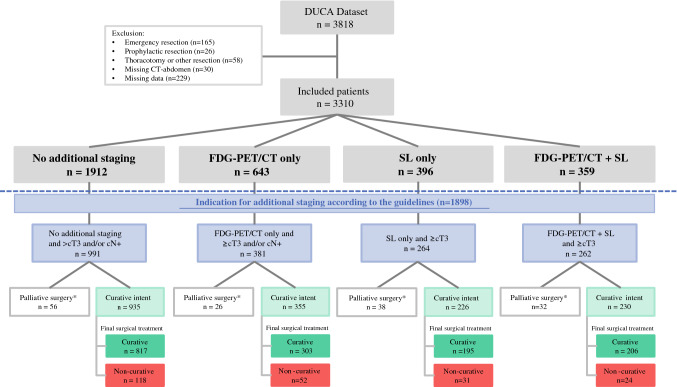
Study flowchart. For analysis on the number of non-curative surgeries, only patients with a curative intent of treatment, as determined prior to surgery, were included (green outline). Final treatment represents the final treatment that has taken place, as determined at the end of surgery. * As determined prior to surgery. *CT* computed tomography, *FDG*-*PET/CT* ^18^F-fluorodeoxyglucose positron emission tomography with computed tomography, *SL* staging laparoscopy

In The Netherlands, 3818 patients underwent surgery for gastric adenocarcinoma in the period 2011–2018. A total of 508 patients were excluded due to an emergency setting (*n* = 165), prophylactic (*n* = 26) or other resection, other than gastrectomy (*n* = 58), missing CT scans (*n* = 30), missing time points (*n* = 100), or missing data (*n* = 129). Of the remaining 3310 patients, 1912 did not undergo either FDG-PET/CT or laparoscopy, 643 patients underwent FDG-PET/CT only, 396 patients underwent solely SL, and 359 patients underwent both diagnostic modalities (Fig. 1). Baseline characteristics are presented in Table 1. Patients undergoing FDG-PET/CT had more comorbidities compared with the other groups. Patients undergoing SL were younger, had a marginally lower body mass index (BMI) and a more favorable American Society of Anesthesiologists (ASA) classification. Patients undergoing the diagnostic modalities were more frequently referred to a high-volume center and had more advanced tumors.

**Table 1:** Baseline characteristics of 3310 patients who underwent surgery for gastric cancer

	No additional staging modalities [*n* = 1912]	FDG-PET/CT [*n* = 643]	Staging laparoscopy [*n* = 396]	FDG-PET/CT and staging laparoscopy [*n* = 359]	*p* value	Missing values (%)
*Patient characteristics*						
Age, years (mean ± SD)	69.9 ± 11.7	69.7 ± 11.2	65.9 ± 11.7	66.8 ± 11.1	< 0.001	0 (0)
BMI, kg/m^2^ (mean ± SD)	25.4 ± 4.6	25.6 ± 4.3	24.7 ± 4.0	24.9 ± 4.4	0.003	63 (2)
Sex					0.289	0 (0)
Male	1189 (62)	421 (66)	240 (61)	217 (60)		
Female	723 (38)	222 (35)	156 (39)	142 (40)		
ASA classification					0.008	17 (1)
I–II	1270 (67)	418 (65)	296 (75)	251 (70)		
III–IV	629 (33)	221 (35)	100 (25)	108 (30)		
Comorbidities	1557 (81)	544 (85)	308 (78)	300 (84)	0.035	0 (0)
Cardiac^a^	609 (32)	232 (36)	85 (22)	103 (29)	< 0.001	0 (0)
Vascular^b^	794 (42)	285 (44)	151 (38)	150 (42)	0.270	0 (0)
Diabetes mellitus	354 (19)	113 (18)	59 (15)	69 (19)	0.339	0 (0)
Pulmonary^c^	315 (17)	121 (19)	44 (11)	59 (16)	0.013	0 (0)
Malignancy^d^	302 (16)	147 (23)	61 (16)	67 (19)	0.001	65 (2)
Previous abdominal or thoracic surgery	770 (40)	280 (44)	150 (38)	142 (40)	0.292	5 (< 1)
*Tumor characteristics*						
cT stage					< 0.001	0 (0)
< cT3	583 (42)	183 (38)	77 (23)	5818 (30)		
≥ cT3^e^	810 (58)	303 (62)	264 (77)	262 (82)		
cTx	770 (23)	196 (20)	94 (13)	676 (27)		
cN stage					< 0.001	0 (0)
N0	1045 (63)	312 (56)	162 (45)	137 (40)		
N+	627 (38)	249 (44)	197 (55)	208 (60)		
Nx	373 (11)	96 (10)	51 (7)	322 (13)		
cM stage					< 0.001	0 (0)
M0	1790 (99)	594 (67)	347 (93)	317 (91)		
M1	22 (1)	20 (3)	28 (8)	32 (9)		
Mx	160 (5)	39 (4)	31 (4)	129 (5)		
Tumor location					< 0.001	41 (1)
Fundus	104 (6)	78 (12)	29 (7)	38 (11)		
Corpus	577 (31)	213 (34)	148 (37)	128 (36)		
Antrum	858 (46)	226 (36)	139 (35)	127 (36)		
Pylorus	154 (8)	46 (7)	33 (8)	28 (8)		
Whole stomach	89 (5)	43 (7)	40 (10)	26 (7)		
Residual stomach	97 (5)	30 (5)	7 (2)	26 (7)		
*Referral status*					< 0.001	280 (9)
Diagnosis in the treatment hospital	635 (37)	168 (29)	108 (28)	69 (20)		
Diagnosis in another hospital	1075 (63)	420 (71)	274 (72)	281 (80)		
*Hospital volume*					< 0.001	0 (0)
< 20 gastrectomies	535 (28)	122 (19)	41 (10)	20 (6)		
20–40 gastrectomies	734 (38)	269 (42)	174 (44)	137 (38)		
> 40 gastrectomies	643 (34)	252 (39)	181 (46)	202 (56)		

Data are expressed as *n* (%) unless otherwise specified

Percentages may not add up to 100% due to rounding

*ASA* American Society of Anesthesiologists; *BMI* body mass index; *FDG*-*PET/CT* ^18^F-fluorodeoxyglucose positron emission tomography with computed tomography; *SD* standard deviation; *PTCA* percutaneous transluminal coronary angioplasty; *CABG* coronary artery bypass graft

^a^Patients with a history of angina pectoris, myocardial infarction, congestive heart failure, PTCA, CABG, valve insufficiency or replacement, heart rhythm disorders, cardiomyopathy, status after heart transplant

^b^Patients with hypertension of peripheral vascular disease

^c^Patients with asthma or chronic obstructive pulmonary disease

^d^Currently or previously treated malignancy other than gastric carcinoma

^e^Of whom 1495 patients underwent curative treatment

The majority of patients underwent neoadjuvant chemotherapy (55%), 50 patients (2%) underwent neoadjuvant chemoradiotherapy, 3 patients (< 1%) underwent neoadjuvant radiotherapy, and 44% of patients did not undergo neoadjuvant treatment. In total, 86% of patients underwent curative surgery, 4% underwent palliative surgery, and in 10% no resection was performed (a futile procedure in 7% and construction of a bypass in 3%). Total gastrectomy was performed in 1235 patients (37%), and subtotal gastrectomy was performed in 1749 patients (53%). The mean (± standard deviation [SD]) waiting time from diagnosis to the start of treatment for all patients was 36 days (± 18.9) for neoadjuvant treatment and 54 days (± 31.7) for primary surgery.

## ^18^F-Fluorodeoxyglucose Positron Emission Tomography with Computed Tomography

**Fig. 2:**
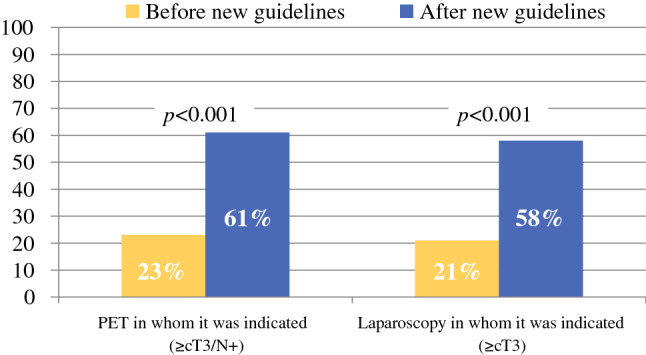
Use of FDG-PET/CT and SL before and after implementation of the revised national guidelines. *FDG*-*PET/CT* ^18^F-fluorodeoxyglucose positron emission tomography with computed tomography, *SL* staging laparoscopy

Before implementation of the guidelines, FDG-PET/CT was performed in 323/1389 patients (23%) for whom this would have been indicated according to the revised guidelines (≥ cT3 and/or N + tumors), whereas after implementation of the guidelines 354/583 patients (61%) underwent FDG-PET/CT (*p* < 0.001) [Fig. 2]. However, after implementation of the guidelines, the use of FDG-PET/CT also increased in patients in whom it was not recommended by these guidelines (17% vs. 43%).

Regarding waiting times, multivariable linear regression analyses showed that FDG-PET/CT was associated with an additional waiting time of 4 days (*p* < 0.001) in the patients who were treated with neoadjuvant chemotherapy. In the group of patients who underwent primary surgery, FDG-PET/CT was associated with 20 extra waiting days (*p* < 0.001). These results are presented in Tables 2 and 3.

**Table 2:** Waiting time to neoadjuvant treatment

	Univariable Mean waiting time (days, SD)	Multivariable^a^			
		B^b^	95% CI	Additional days	*p* value
*Modalities*					
None	28.9 (± 15.9)	Ref	–	–	–
FDG-PET/CT	36.0 (± 18.5)	0.19	0.07–0.31	4	0.001
Staging laparoscopy	37.5 (± 15.0)	0.34	0.24–0.45	8	< 0.001
Both modalities	47.0 (± 23.9)	0.52	0.41–0.62	14	< 0.001

Multivariable linear regression analyses on the influence of patient, tumor, and diagnostic characteristics on waiting time from diagnosis to the start of neoadjuvant treatment (*n* = 1808)

^a^Adjusted for age, BMI, weight loss, comorbidities (overall, cardiac, pulmonary, previous malignancy, previous abdominal surgery), ASA, referral status, location of tumor, cT stage, cN stage, hospital volume

^b^Intercept = 3.008 (20 days)

*ASA* American Society of Anesthesiologists; *FDG*-*PET/CT* ^18^F-fluorodeoxyglucose positron emission tomography with computed tomography; *SD* standard deviation; *CI* confidence interval; *BMI* body mass index

**Table 3:** Waiting time to surgical treatment

	Univariable Mean waiting time (days, SD)	Multivariable^a^			
		B^b^	95% CI	Additional days	*p* value
*FDG*-*PET/CT*					
No	50.2 (± 28.3)	Ref	–	–	–
Yes	65.1 (± 38.2)^c^	0.28	0.20–0.36	20	< 0.001

Multivariable linear regression analyses on the influence of patient, tumor, and diagnostic characteristics on waiting time from diagnosis to primary surgical treatment with curative intent (*n* = 1332)

^a^Adjusted for age, BMI, weight loss, comorbidities (overall, cardiac, pulmonary, previous malignancy, previous abdominal surgery), ASA, referral status, location of tumor, cT stage, cN stage, hospital volume

^b^Intercept = 4.129 (62 days)

^c^Significantly different

*ASA* American Society of Anesthesiologists; *FDG*-*PET/CT* ^18^F-fluorodeoxyglucose positron emission tomography with computed tomography; *SD* standard deviation; *CI* confidence interval; *BMI* body mass index

### Staging Laparoscopy

Before implementation of the revised guidelines, SL was performed in 237/1140 patients (21%) in whom this would have been indicated according to the revised guidelines (≥ cT3 tumors). This percentage increased to 58% (289/499, *p* < 0.001) after implementation of the revised guidelines (Fig. 2). Additionally, the use of SL also increased in patients in whom there was no indication according to the revised guidelines, after its implementation (from 8 to 32%).

SL was associated with an additional waiting time of 8 days (*p* < 0.001) to the start of neoadjuvant chemotherapy in the group of patients who received neoadjuvant chemotherapy (Table 2).

In all patients who underwent either solely SL (*n* = 396) or both modalities (*n* = 359), SL identified metastases or irresectable disease in 76 patients (10%, numbers not shown in Fig. 1), resulting in a preoperatively determined palliative intent in these patients. In the group of patients who had not undergone either FDG-PET/CT or SL (*n* = 1912), a palliative intent of treatment was registered in 67 patients (4%), versus 36 patients (6%) in the group of patients who underwent solely FDG-PET/CT (*n* = 643).

### Non-curative Surgery

For analyzing the effects of FDG-PET/CT and SL on the number of non-curative resections as determined at the end of the procedure, only those patients with at least a cT3 and/or N + tumor and curative intent were selected. A total of 1746 patients with at least a cT3 and/or N + tumor were treated with curative intent (Fig. 1). Of these, 225 patients (13%) were eventually registered at the end of the surgical procedure as having undergone non-curative surgery, consisting of 51 patients in whom a palliative resection had been performed, 57 patients who received a bypass (i.e. no resection), and 117 patients who underwent a futile procedure. The incidence of intraoperatively determined non-curative surgery did not differ between the patient groups undergoing one or both of the staging modalities (13% after none; 15% after FDG-PET/CT; 14% after SL; 10% after both modalities; *p* = 0.492).

## Discussion

In this population-based study, the implementation of FDG-PET/CT and SL for patients with gastric cancer in The Netherlands, and their association with logistics and the proportion of non-curative procedures, were evaluated. After revision of the national guidelines in July 2016, which now recommend FDG-PET/CT and SL in patients with locally advanced tumors, significantly more FDG-PET/CTs and SLs were performed. Remarkably, the increase in PET/CT and SL was not only observed in patients with an indication for these modalities according to the guidelines but also in patients without a predefined indication. This may be due to treating physicians becoming more aware of the possible value of FDG-PET/CT and SL, and therefore also requesting these procedures in other patients who they regard at increased risk for metastases. Referral to a high-volume center more frequently resulted in performing FDG-PET/CT and SL, which was associated with a significantly longer waiting time from diagnosis to the start of treatment. Performing SL or both modalities consecutively may not be associated with the incidence of non-curative surgery.

Although FDG-PET/CT and SL were more frequently performed for locally advanced gastric tumors, approximately 40% of the patients in whom this was indicated still did not undergo FDG-PET/CT and SL in the current study. This might be explained by a lag time between publication of guidelines and their adoption in clinical practice.^15^ Several general barriers for the adoption of new guidelines have been identified and reported, such as lack of awareness, lack of agreement with the new guidelines, and lack of outcome expectancy.^15^ Interventions to promote the implementation of research findings include educational outreach visits.^16^ As part of the PLASTIC study,^17^ a prospective observational cohort study in The Netherlands that evaluated the impact and cost effectiveness of FDG-PET/CT and SL in addition to initial staging (CT and gastroscopy) in patients with locally advanced gastric cancer, these educational visits started in August 2017. Other factors that have been reported to contribute to slow implementation are the qualities of the guidelines (such as compatibility with existing beliefs and values, or complexity), characteristics of the health care practice setting (including legal and financial aspects), and characteristics of the healthcare professional (e.g. age).^18^ Besides delayed adoption of the revised guidelines in clinical practice, there might be other factors contributing to not performing FDG-PET/CT or SL in appropriate patients. The general reasons for refraining from SL may include older age (as older patients are frailer and have more comorbidities), tumors causing complications (e.g. obstruction, hemorrhage, perforation), and a history of prior upper abdominal surgery with severe adhesions.^19^,^20^

In the current study, 32–43% of patients underwent FDG-PET/CT or SL, although there was no indication according to the current guidelines. In this context, it is important to note that clinical staging of gastric cancer is known to be inaccurate.^5^,^6^,^21^^–^23 Several reasons to perform additional diagnostics in patients with lower tumor stages may exist, such as excessive weight loss or previous malignancy, which might increase the clinical suspicion of occult metastases. Nevertheless, considering additional diagnostics in patients for whom there is no accepted indication according to guidelines should be performed with care as longer waiting times impair quality of life and might allow for tumor progression. Other possible disadvantages include higher diagnostic health care costs, incidental findings that require further investigations, and possible morbidity due to SL. To slightly elaborate on the costs, an FDG-PET/CT costs €1200 on average^24^ and an SL costs €900 on average, based on the minute price of the operating room (including operating room, nurses, surgeon, anesthesiologist, overheads).^25^ However, we await the results of the PLASTIC study in order to make statements on the economic aspects.

Although literature on whether or not high-volume centers follow directives more frequently is not available, the current study concluded that FDG-PET/CT and SL were more frequently performed in higher-volume centers. It has been previously reported that centralization of gastric cancer care in high-volume centers in The Netherlands resulted in improved postoperative outcomes.^11^,^26^ The results of the current study confirm that the referral of patients to high-volume centers may result in better health care by providing clinical care in accordance with the guidelines.

Baseline waiting times found in the current study fell within the indicated waiting times advised and aimed at by Dutch guidelines.^27^ In addition, baseline waiting times were comparable with previously reported median waiting times of 4.6 weeks to the start of neoadjuvant treatment and 6 weeks to primary surgery.^28^ Performing FDG-PET/CT or SL was associated with a significantly prolonged waiting time from diagnosis to the start of treatment, both for neoadjuvant treatment (although clinically less relevant) and primary surgical treatment. Patients undergoing primary surgery are usually older and have several comorbidities, and are therefore not deemed fit enough for chemotherapy.^29^,^30^ It is possible that in these patients, additional findings are more frequently detected on FDG-PET/CT or during SL, for which further diagnostics are required. Other confounding factors might also contribute to increasing waiting times. For example, generally increasing waiting times due to pressure on the health care system and centralization of gastric cancer treatment might play a role as patients had to be referred to tertiary centers more often over the years. Regardless of the potential causes, it is questionable what the clinical relevance of the reported extended waiting times is, since previous studies suggested that an additional waiting time of some weeks does not lead to decreased long-term survival.^28^

Smyth et al.^8^ conducted a study of 113 locally advanced gastric cancer patients (cT3-4) and reported a 10% reduction in the number of futile procedures after performing an FDG-PET/CT, and a decrease of 19% after SL. Findlay et al.^31^ performed a study of 279 gastric cancer patients and reported unsuspected metastases found with FDG-PET/CT in 7% of patients. In the study from Bosch et al.,^32^ additional metastases were detected in 16% of 105 patients. These findings on FDG-PET/CT resulted in a treatment change from curative to palliative intent, and prevention of futile surgery with accompanying morbidity in these patients. Unfortunately, as the DUCA does not register patients in whom surgery was omitted based on findings on FDG-PET/CT, the results of our study can neither confirm nor refute these results. Regarding the detection of metastatic or irresectable disease in the case of SL, literature on the yield of SL varies from detection rates of 19–52%. Furthermore, the percentage found in this study (10%) does not completely support these previously published numbers.^8^,^33^^–^35 An explanation for this might be that peritoneal lavage is also included in the aforementioned studies and scored as positive SL, whereas in the current dataset, neither information on whether peritoneal lavage has been performed nor outcomes of the SL are registered. In our study, no differences were found in the number of intraoperatively determined non-curative procedures when comparing the performance of no, solely one, or both staging modalities; however, it should be noted that this was analyzed during the implementation phase of the guideline.

Several other limitations apply to the current study. First, no data on the outcomes of FDG-PET/CT are available and patients not undergoing surgery are not registered in the DUCA as it is a surgical registry, which could have resulted in underestimation of the proportions reported in this study. Therefore, it is not possible to draw firm conclusions on the treatment changes based on FDG-PET/CT findings. Second, the dataset used for this study does not contain histopathology data, while several studies report that FDG-PET/CT may specifically be useful in patients with specific tumor biology or characteristics, such as intestinal type or poorly differentiated adenocarcinomas.^6^,^23^ For these reasons, the results of the PLASTIC trial, also evaluating histopathology data, are awaited.^17^

## Conclusion

This population-based study demonstrates that FDG-PET/CT and SL have increasingly been used in patients with locally advanced gastric tumors in The Netherlands, mainly in high-volume centers, at the expense of prolonged waiting times from diagnosis to the start of treatment. No differences in the proportion of non-curative procedures were found when performing SL or both modalities consecutively in the patients who had an indication. However, it should be noted that no firm conclusions can be made on solely performing FDG-PET/CT, and therefore the results of the PLASTIC study should be awaited. Future studies should focus on patient selection for FDG-PET/CT and SL and the potential consequences of prolonged waiting times.

## References

[1] Integraal Kankercentrum Nederland. Diagnostiek, behandeling en follow-up van het maagcarcinoom. 2016. pp. 1–7. http://www.oncoline.nl/uploaded/docs/Maagcarcinoom/Richtlijnmaagcarcinoom.pdf. Accessed 1 June 2019.

[2] Cunningham D, Allum WH, Stenning SP, Thompson JN, van de Velde CJH, Nicolson M (2006). Perioperative chemotherapy versus surgery alone for resectable gastroesophageal cancer. N Engl J Med.

[3] Al-Batran SE, Homann N, Pauligk C, Goetze TO, Meiler J, Kasper S (2019). Perioperative chemotherapy with fluorouracil plus leucovorin, oxaliplatin, and docetaxel versus fluorouracil or capecitabine plus cisplatin and epirubicin for locally advanced, resectable gastric or gastro-oesophageal junction adenocarcinoma (FLOT4): a randomised, phase 2/3 trial. Lancet.

[4] Integraal Kankercentrum Nederland. Richtlijn Diagnostiek, behandeling en follow-up van het maagcarcinoom 2009.

[5] Choi JY, Shim K-N, Kim S-E, Jung H-K, Jung S-A, Yoo K (2014). The Clinical Value of 18F-Fluorodeoxyglucose Uptake on Positron Emission Tomography/Computed Tomography for Predicting Regional Lymph Node Metastasis and Non-curative Surgery in Primary Gastric Carcinoma. Korean J Gastroenterol..

[6] Seevaratnam R, Cardoso R, McGregor C, Lourenco L, Mahar A, Sutradhar R (2012). How useful is preoperative imaging for tumor, node, metastasis (TNM) staging of gastric cancer? A meta-analysis. Gastric Cancer.

[7] Wang Z, Chen J-Q (2011). Imaging in assessing hepatic and peritoneal metastases of gastric cancer: a systematic review. BMC Gastroenterol.

[8] Smyth E, Schöder H, Strong VE, Capanu M, Kelsen DP, Coit DG (2012). A prospective evaluation of the utility of 2-deoxy-2-[^18^F]fluoro-d-glucose positron emission tomography and computed tomography in staging locally advanced gastric cancer. Cancer.

[9] Busweiler LAD, Wijnhoven BPL, van Berge Henegouwen MI, Henneman D, van Grieken NCT, Wouters MWJM (2016). Early outcomes from the Dutch Upper Gastrointestinal Cancer Audit. Br J Surg.

[10] Washington K (2010). 7th edition of the AJCC cancer staging manual: Stomach. Ann Surg Oncol.

[11] Haverkamp L, Ruurda JP, van der Sluis PC, van Hillegersberg R (2013). Chirurgische behandeling van maagcarcinoom: Focus op centralisatie en laparoscopische resecties. Ned Tijdschr Geneeskd.

[12] Nelen SD, Heuthorst L, Verhoeven RHA, Polat F, Kruyt PM, Reijnders K (2017). Impact of Centralizing Gastric Cancer Surgery on Treatment, Morbidity, and Mortality. J Gastrointest Surg.

[13] Sano T, Kodera Y (2011). Japanese classification of gastric carcinoma: 3rd English edition. Gastric Cancer.

[14] Cunningham D, Starling N, Rao S, Iveson T, Nicolson M, Coxon F (2008). Capecitabine and Oxaliplatin for Advanced Esophagogastric Cancer. N Engl J Med.

[15] Cabana MD, Rand CS, Powe NR, Wu AW, Wilson MH, Abboud P-AC (1999). Why don’ t physicians follow a framework for improvement. JAMA..

[16] Bero LA, Grilli R, Grimshaw JM, Harvey E, Oxman AD, Thomson MA (1998). Closing the gap between research and practice: an overview of systematic reviews of interventions to promote the implementation of research findings. Br Med J.

[17] Brenkman HJF, Gertsen EC, Vegt E, van Hillegersberg R, van Berge Henegouwen MI, Gisbertz SS (2018). Evaluation of PET and laparoscopy in STagIng advanced gastric cancer: a multicenter prospective study (PLASTIC-study). BMC Cancer.

[18] Davis DA, Taylor-Vaisey A (1997). A systematic review of theoretic concepts, practical experience and research evidence in the adoption of clinical practice guidelines. Can Med Assoc J.

[19] Machairas N, Charalampoudis P, Molmenti EP, Kykalos S, Tsaparas P, Stamopoulos P (2017). The value of staging laparoscopy in gastric cancer. Ann Gastroenterol..

[20] Society of American Gastrointestinal and Endoscopic Surgeons. Guidelines for diagnostic laparoscopy, vol. 13. Society of American Gastrointestinal and Endoscopic Surgeons; 1999. 10.1007/s004649900944. 9918636

[21] Kwee RM, Kwee TC (2009). Imaging in assessing lymph node status in gastric cancer. Gastric Cancer.

[22] Borggreve AS, Goense L, Brenkman HJF, Mook S, Meijer GJ, Wessels FJ (2019). Imaging strategies in the management of gastric cancer: Current role and future potential of MRI. Br J Radiol.

[23] Kwee RM, Kwee TC (2007). Imaging in local staging of gastric cancer: a systematic review. J Clin Oncol.

[24] Dutch Healthcare Authority (Nederlandse Zorgautoriteit). Performance and Tariffs Specialist Medical Care (Prestaties en tarieven medisch-specialistische zorg). Dutch Healthcare Authority; 2017.

[25] Bolkenstein HE, de Wit GA, Consten ECJ, Van de Wall BJM, Broeders IAMJ, Draaisma WA (2019). Cost-effectiveness analysis of a multicentre randomized clinical trial comparing surgery with conservative management for recurrent and ongoing diverticulitis (DIRECT trial). Br J Surg.

[26] Busweiler LAD, Dikken JL, Henneman D, van Berge Henegouwen MI, Ho VKY, Tollenaar RAEM (2017). The influence of a composite hospital volume on outcomes for gastric cancer surgery: a Dutch population-based study. J Surg Oncol.

[27] Stichting Oncologische Samenwerking (2015). Multidisciplinaire Normering Oncologische Zorg in Nederland. PodoPost.

[28] Brenkman HJF, Visser E, van Rossum PSN, Siesling S, van Hillegersberg R, Ruurda JP (2017). Association Between Waiting Time from Diagnosis to Treatment and Survival in Patients with Curable Gastric Cancer: A Population-Based Study in the Netherlands. Ann Surg Oncol.

[29] Sheng S, Chen Y, Li C (2018). Outcomes of laparoscopic total gastrectomy for elderly gastric cancer patients. J Cancer.

[30] Saif MW, Makrilia N, Zalonis A, Merikas M, Syrigos K (2010). Gastric cancer in the elderly: An overview. Eur J Surg Oncol.

[31] Findlay JM, Antonowicz S, Segaran A, el Kafsi J, Zhang A, Bradley KM (2019). Routinely staging gastric cancer with ^18^F-FDG PET-CT detects additional metastases and predicts early recurrence and death after surgery. Eur Radiol.

[32] Bosch KD, Chicklore S, Cook GJ, Davies AR, Kelly M, Gossage JA (2020). Staging FDG PET-CT changes management in patients with gastric adenocarcinoma who are eligible for radical treatment. Eur J Nucl Med Mol Imaging.

[33] Ikoma N, Blum M, Chiang YJ, Estrella JS, Roy-Chowdhuri S, Fournier K (2016). Yield of Staging Laparoscopy and Lavage Cytology for Radiologically Occult Peritoneal Carcinomatosis of Gastric Cancer. Ann Surg Oncol.

[34] Groh EM, Gupta S, Brown ZJ, Enewold L, Gamble LA, Hernandez JM (2020). Staging Laparoscopy is Underutilized in the Management of Gastric Adenocarcinoma. Ann Surg Oncol..

[35] Fukagawa T (2019). Role of staging laparoscopy for gastric cancer patients. Ann Gastroenterol Surg..

